# Advances in functional analysis of the microbiome: Integrating metabolic modeling, metabolite prediction, and pathway inference with Next-Generation Sequencing data

**DOI:** 10.71150/jm.2411006

**Published:** 2025-01-24

**Authors:** Sungwon Jung

**Affiliations:** 1 Department of Genome Medicine and Science, Gachon University College of Medicine, Incheon 21565, Republic of Korea; 2 Gachon Institute of Genome Medicine and Science, Gachon University Gil Medical Center, Incheon 21565, Republic of Korea

**Keywords:** microbiome functional analysis, genome-scale metabolic models, metabolite prediction, pathway activity inference, next-generation sequencing, microbial interactions

## Abstract

This review explores current advancements in microbiome functional analysis enabled by next-generation sequencing technologies, which have transformed our understanding of microbial communities from mere taxonomic composition to their functional potential. We examine approaches that move beyond species identification to characterize microbial activities, interactions, and their roles in host health and disease. Genome-scale metabolic models allow for in-depth simulations of metabolic networks, enabling researchers to predict microbial metabolism, growth, and interspecies interactions in diverse environments. Additionally, computational methods for predicting metabolite profiles offer indirect insights into microbial metabolic outputs, which is crucial for identifying biomarkers and potential therapeutic targets. Functional pathway analysis tools further reveal microbial contributions to metabolic pathways, highlighting alterations in response to environmental changes and disease states. Together, these methods offer a powerful framework for understanding the complex metabolic interactions within microbial communities and their impact on host physiology. While significant progress has been made, challenges remain in the accuracy of predictive models and the completeness of reference databases, which limit the applicability of these methods in under-characterized ecosystems. The integration of these computational tools with multi-omic data holds promise for personalized approaches in precision medicine, allowing for targeted interventions that modulate the microbiome to improve health outcomes. This review highlights recent advances in microbiome functional analysis, providing a roadmap for future research and translational applications in human health and environmental microbiology.

## Introduction

The microbiome, consisting of diverse microorganisms such as bacteria, viruses, fungi, and archaea, is essential for maintaining host health and influencing disease states. Advances in next-generation sequencing (NGS) technologies have transformed microbiome research, enabling deeper exploration of microbial communities across various environments, including the human gut, skin, and soil ecosystems. While traditional sequencing methods like 16S rRNA profiling and metagenomics reveal microbial composition, understanding the functional potential of these communities remains a crucial challenge.

Functional microbiome analysis aims to elucidate how microbial communities contribute to host physiology, metabolism, and disease processes. Key approaches include genome-scale metabolic models (GEMs), which simulate microbial metabolic networks to predict growth rates, metabolic interactions, and responses to environmental changes. GEMs enable researchers to characterize metabolic capabilities of individual microorganisms or communities, providing insights into microbial functions in diverse contexts.

Another promising approach is the prediction of metabolite profiles from metagenomic or metatranscriptomic data. Computational tools, including machine learning models, leverage microbial gene abundances to infer potential metabolic outputs, which can identify metabolites relevant to host health and disease. Additionally, pathway activity inference methods use microbial data to assess metabolic processes within microbial communities, revealing responses to environmental or pathological conditions.

These approaches—GEMs, metabolite prediction, and pathway inference—advance microbiome functional analysis beyond taxonomic descriptions. By integrating these methods with NGS data, researchers can gain a comprehensive understanding of microbial interactions and functions, paving the way for targeted microbiome-based interventions in health and environmental applications.

## Glossary of Technical Terms

*Amplicon sequence variant (ASV):* Highly resolved DNA sequences used in microbial community profiling to distinguish between closely related organisms

*Constraint-based optimization:* A mathematical approach used to find optimal solutions under a set of defined constraints, widely applied in GEMs

*Dynamic flux balance analysis (dFBA):* An extension of flux balance analysis (FBA) that incorporates time-dependent changes in metabolite concentrations to simulate dynamical biological processes

*F1 score:* The harmonic mean of precision and recall, providing a balance between these two metrics for evaluating model performance

*FBA:* A constraint-based optimization method that can be used in GEMs to predict the flow of metabolites through metabolic networks

*GEM:* Mathematical model that simulates the metabolic capabilities of individual organism or communities based on their genomic content

*Hidden Markov model (HMM):* A statistical model used to represent sequences of observed data, often applied in functional annotation and sequence alignment

*Metagenomics:* The study of genetic material recovered directly from environmental samples, providing insights into the composition and functions of microbial communities

*Microbiome:* The collective genomes of microorganisms such as bacteria, fungi, viruses, and archaea that reside in a specific environment

*NGS:* Advanced sequencing technologies that allow rapid sequencing of entire genomes or targeted regions, enabling detailed analysis of microbiomes

*Objective function:* A mathematical expression used in optimization problems, representing a goal (e.g., maximizing growth or metabolite production) in metabolic models

*Pathway activity inference:* Methods that analyze microbial data to assess functional metabolic processes within microbial communities

*Phylogenetic function inference:* A method used to predict the functional capabilities of microorganisms based on their evolutionary relationships

*Precision:* A measure of a predictive model’s accuracy in identifying true positives out of all predicted positives

*Recall:* A measure of a model’s ability to identify all true positives from the actual positive cases

*Stoichiometric coefficient of reaction:* A numerical value that represents the proportion of each reactant and product involved in a chemical reaction, crucial in defining metabolic models

## GEMs for Microbiome Research

**Fig. 1.**
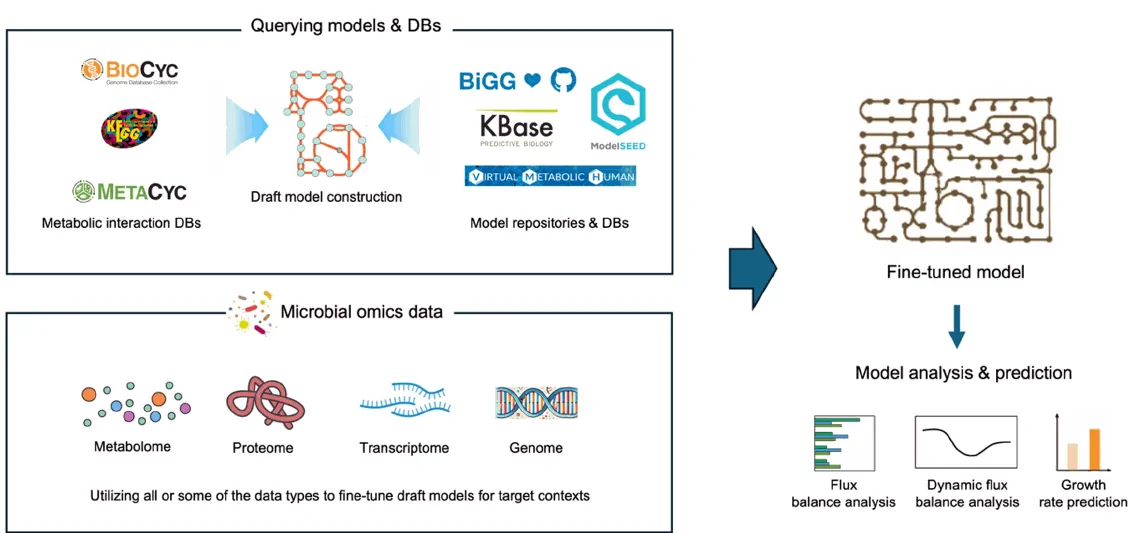
The general workflow of building and using GEMs. Draft models are built based on known metabolic interactions and pre-built models, then fine-tuned based on context-specific omics profiles. Final models can be used for various purposes including the prediction of metabolite fluxes (FBA and dFBA) and the prediction of microbial growth based on metabolic activity.

The human microbiome plays a critical role in health and disease, and its study has been significantly advanced by the development of GEMs. These models provide a framework for simulating the metabolic capabilities of individual microbes and microbial communities within the microbiome. GEMs facilitate the integration of various types of omics data and allow researchers to infer the metabolic interactions between the microbiota and its host, as well as within microbial communities themselves (Fig. 1). In this section, we provide an overview of the theoretical foundations of GEMs, related tools, repositories, microbial community models, and applications in microbiome research. An overview of GEM tools and resources is listed in Table 1, and the representative studies are described in the following subsections.

### Theoretical foundations of GEMs

GEMs are powerful tools for studying metabolic networks at both the single-species and community levels. These models rely on a set of mathematical and theoretical principles that define how metabolic fluxes are distributed under various physiological conditions. There are fundamental assumptions and mathematical frameworks that underpin GEMs, including their most widely used analytical method – FBA.

*Steady-state assumption:* One of the core assumptions in GEMs is that the metabolic network operates at a steady state, where the concentration of intracellular metabolites remains constant over time. This is mathematically expressed as:


(1) S·v=0

, where *S* is the stoichiometric matrix, representing the stoichiometric coefficients of metabolites in each reaction. v is the flux vector, representing the rate of each reaction in the network. The steady-state assumption simplifies the system by focusing on the balance of metabolic fluxes, rather than the dynamics of metabolite concentrations.

*FBA:* FBA is a constraint-based optimization method that predicts the distribution of metabolic fluxes by maximizing or minimizing an objective function, typically representing cellular growth or metabolite production. The optimization problem is formulated as:


(2) $$\text{maximize}\;Z=\sum_{i=1}^{n}c_{i}v_{i}$$

with the vector of fluxes v (to be determined), while satisfying *S* ∙ *v* = 0 and *v*_min_ ≤*v* ≤ *v*_max_. *Z* is the objective function value, *c_i_* is the coefficient representing the contribution of flux *v_i_* to the objective function, *v*_min_ and *v*_max_ are the lower and upper bounds of fluxes, respectively. By computing the optimal v, FBA can provide insights into the metabolic capabilities of organisms under specific environmental conditions and genetic configurations.

*dFBA:* While FBA assumes a static steady-state, dFBA incorporates temporal dynamics by modeling changes in metabolite concentrations over time. This approach uses ordinary differential equations to describe the time evolution of extracellular metabolite concentrations:


(3) $$\frac{dC}{dt}=S_{ext}\cdot v$$

, where *C* represents extracellular metabolite concentrations and *S*_ext_ is the stoichiometric matrix for extracellular metabolites, thus explaining the dynamics of extracellular metabolites with the dynamics of the metabolic network. dFBA is valuable for simulating time-dependent process such as growth dynamics and environmental changes.

*Assumptions and limitations:* These analysis methods of GEMs rely on specific assumptions and have limitations. FBA assumes optimal behavior of the system, which may not always align with real biological systems. dFBA requires more computational resources and detailed knowledge of initial metabolite concentrations. Despite these limitations, GEMs provide a robust framework for simulating and analyzing metabolic networks, offering insights into microbial behavior and metabolic interactions.

### GEM modeling tools

GEMs allow for the simulation of metabolic networks based on genomic, transcriptomic, proteomic, and metabolomic data integration, facilitating in-depth study of microbial metabolic capabilities. Widely used GEM tools include the COBRA toolbox (Heirendt et al., 2019), a MATLAB-based suite for constraint-based modeling, which enables the construction of models for individual microbes and communities, supporting analyses like FBA to predict growth, metabolite production, and environmental responses.

Other popular tools, such as the RAVEN toolbox (Wang et al., 2018), focus on reconstructing metabolic models and performing flux analyses, with applications in diet-microbiome studies and large-scale community modeling. CarveMe (Machado et al., 2018), streamlines GEM construction by starting with a universal model that is customized based on genetic evidence, allowing for rapid species-specific model generation. This top-down approach makes CarveMe especially suitable for creating large-scale model libraries with minimal manual curation.

For modeling microbial communities, BacArena (Bauer et al., 2017) and COMETS (Dukovski et al., 2021) provide tailored functionality. BacArena simulates individual metabolic interactions in shared environments, while COMETS allows for dynamic modeling of microbial consortia using stoichiometric approaches. These tools are valuable for studying microbial cooperation, competition, and metabolic cross-feeding within complex communities.

### GEM model repositories and databases

Several model repositories provide access to curated GEMs, enabling researchers to utilize pre-built models or modify them for specific purposes. The BiGG models database (King et al., 2016) is one of the largest repositories, offering high-quality GEMs for a wide range of organisms, including key members of the human gut microbiota. This resource allows researchers to explore, integrate, and modify models for specific strains or species.

Another valuable resource is AGORA2 (Assembly of Gut Organisms through Reconstruction and Analysis, version 2) (Heinken et al., 2023), which contains metabolic reconstructions of 7,302 microbial strains commonly found in the human gut. The Virtual Metabolic Human (VMH) database (Noronha et al., 2019) also publicly provides metabolic reconstructions, which provides integrated view of both human and microbial metabolism. The models via these repositories provide a comprehensive foundation for studying microbe-microbe and host-microbiome interactions, and they have been integrated with host GEMs, such as Recon2 (Swainston et al., 2016) and Recon3D (Brunk et al., 2018), to facilitate host-microbiome metabolic analysis.

In addition to these repositories, other resources such as ModelSEED (Seaver et al., 2021) and KBase (Arkin et al., 2018) offer web-based platforms for GEM reconstruction and analysis, supporting automated model generation from genomic data. These repositories and tools provide critical infrastructure for advancing GEM-based microbiome research.

### GEM of microbial communities

Modeling microbial communities presents unique challenges compared to single-species modeling due to the complexity of interactions between different species and the diverse environmental conditions they inhabit. GEMs of microbial communities are typically constructed by combining GEMs of individual species into a unified framework, where metabolic exchanges occur between species via shared extracellular compartments. This framework facilitates studying interspecies interactions in response to various environmental changes, such as dietary shifts (Kumar et al., 2018; Shoaie et al., 2015; Shoaie & Nielsen, 2014). However, integrating such interactions within a community requires not only the combination of individual models but also approaches that dynamically account for species-specific activity based on contextual data.

Recent tools, such as the Microbiome Modelling Toolbox 2.0 (Heinken & Thiele, 2022), enable high-throughput, personalized microbiome model reconstruction from microbial composition data and include modules for exploring microbe-microbe interactions and the metabolic roles of individual species within a community. Tools like CarveMe (Machado et al., 2018) also have streamlined the process of reconstructing GEMs by using a top-down approach that automatically remove reactions not relevant to a target organism. By leveraging dynamic modules, these tools facilitate large-scale simulations, enhancing the capacity to study community-wide metabolic outcomes under various conditions.

In addition to the basic modeling of microbiome, advanced community GEMs have been employed to explore complex metabolic interactions, including competition, commensalism, and mutualism. For example, pairwise modeling of gut microbes has revealed how multiple microbial species cooperate to produce key metabolites like butyrate, a short-chain fatty acid crucial for gut health (Kumar et al., 2018). Tools like metaGEM (Zorrilla et al., 2021) extend this capacity by reconstructing GEMs directly from metagenomic data, allowing for a more accurate reflection of the metabolic diversity present in microbiomes. To model spatial and temporal aspects of microbial interactions, platforms such as COMETS (Dukovski et al., 2021) integrate GEMs with dFBA in a spatially explicit manner. Individual-based modeling approaches, like those used in BacArena (Bauer et al., 2017), complement GEMs by focusing on the spatially resolved metabolic activities of individual cells within microbial communities. Through this approach, GEMs can represent interspecies metabolic variability and enable dynamic analysis of microbial communities without reliance on reference genomes, which often overlook strain-level functional differences.

## Predicting Metabolite Profiles from Microbiome Sequencing Data

**Fig. 2.**
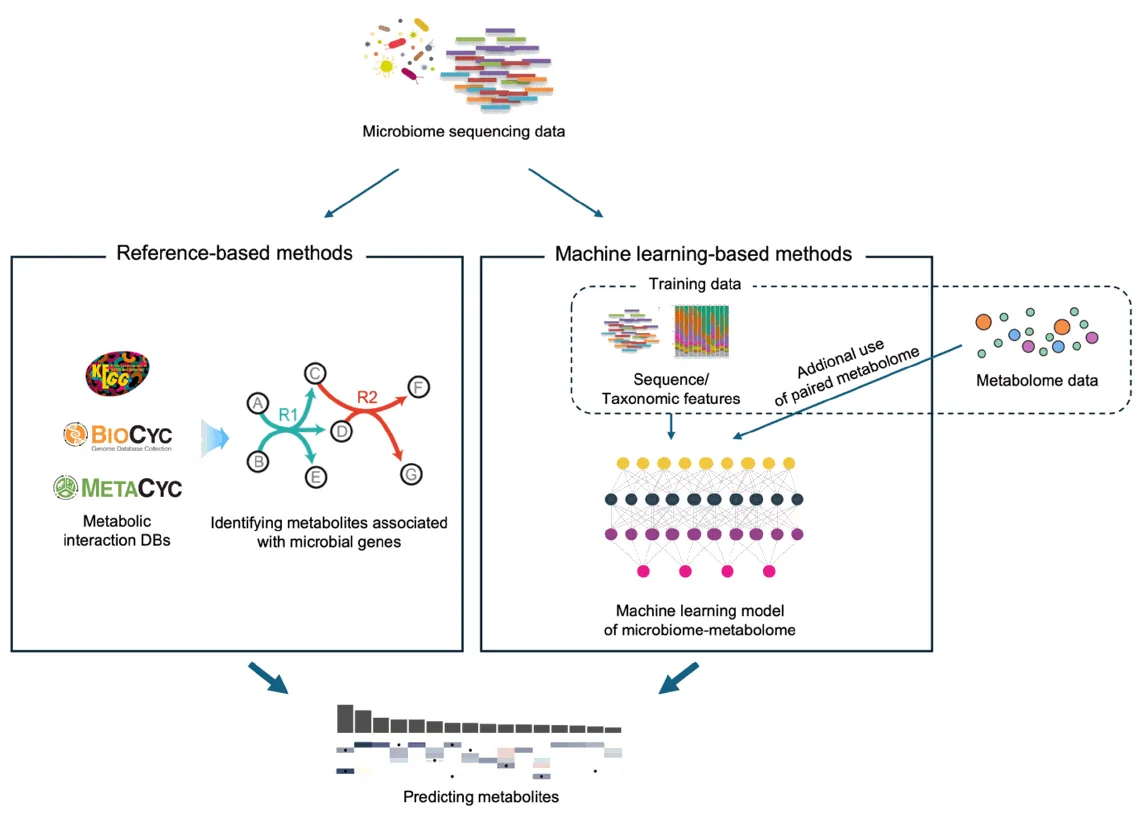
Comparing the approaches of reference-based and ML-based metabolite prediction methods. Reference-based methods utilize known metabolic interactions as well as gene orthologs, while ML-based methods empirically “learn” such associations from training data.

The development of computational tools for predicting metabolite profiles from microbiome sequencing data has emerged as an essential area of study, given the high costs and technical challenges associated with large-scale metabolomics profiling. This predictive approach allows researchers to leverage microbiome sequencing data as a proxy for direct metabolomic measurements, which can inform understanding of microbiome-related health impacts, potential biomarkers, and therapeutic avenues. Recent advances have seen both reference-based and machine learning (ML)-based approaches evaluated for their accuracy and utility in predicting microbial community metabolites, with promising implications for clinical and research applications. In this section, we explore these methodologies, their performance, and associated limitations. Predictive metabolomics relies on computational models that infer the metabolic capabilities of microbial communities based on their genetic content. There are two main categories of methods: Reference-based and ML-based approaches. A brief, summarized comparison of these tools is listed in Table 2 and illustrated in Fig. 2.

### Reference-based approaches

Reference-based methods predict metabolites by mapping microbiome sequencing data onto well-established biochemical databases like KEGG (Kanehisa et al., 2023). BioCyc (Karp et al., 2019), and metabolic pathway-specific databases like MetaCyc (Caspi et al., 2020). These methods rely on curated gene-metabolite associations and enzymatic pathways to infer metabolite profiles. Tools such as Metabolite Identification and Mechanistic Objective-based Systems Analysis 2 (MIMOSA2) (Noecker et al., 2022) and Mangosteen (Yin et al., 2020) follow this approach. MIMOSA2 incorporates reaction networks from databases to model the metabolic potential of microbial communities, accounting for compound flux through microbial gene functions and pathway directionality. Mangosteen uses a similar approach, but with a broader connection to metabolites directly linked to KEGG and BioCyc reactions, facilitating metabolite discovery beyond known metabolic networks.

### ML-based approaches

ML-based methods offer an alternative by learning complex relationships between gene features in microbiome data and metabolite abundance from large, paired microbiome-metabolome datasets. By bypassing predefined pathways, these models are adaptable to previously uncharacterized microbiomes and may better capture host-microbiome interactions. For instance, methods like MiMeNet that leverages machine learning models such as neural networks (Le et al., 2020; Reiman et al., 2021) predict metabolite abundances while facilitating shared learning across metabolites and increasing prediction robustness. MiMeNet further organizes microbes and metabolites into functional modules, revealing patterns of microbe-metabolite interactions with potential clinical relevance. Similarly, MelonnPan (Mallick et al., 2019) has demonstrated accuracy in predicting metabolite presence, although it models each metabolite individually and lacks multivariate learning, which may limit the depth of interactions captured compared to the comprehensive approach of MiMeNet. Building on these advances, LOCATE (Shtossel et al., 2024) offers a more integrated approach by using a latent representation to model the complex bi-directional interactions between microbiome and metabolome, achieving superior prediction accuracy and providing insights into host condition by capturing context-specific microbiome-metabolome dynamics.

### Performance of predictive methods

The accuracy and effectiveness of these predictive tools have been evaluated based on criteria like precision, recall, F1 scores, and the ability to detect differential metabolites between sample groups. A comparative analysis has been conducted using paired microbiome and metabolome data from six studies spanning different diseases, including inflammatory bowel disease (IBD), autism spectrum disorder, and colorectal cancer (Yin et al., 2020).

From comparative evaluation, ML-based methods demonstrated superior performance in predicting the presence or absence of metabolites compared to reference-based approaches, achieving the highest F1 scores across datasets. This improved accuracy is likely attributable to its data-driven design, which can capture complex gene-metabolite interactions not explicitly encoded in reference databases. In contrast, the reference-based tools exhibited higher recall but lower precision, likely due to the broader range of metabolites inferred from multiple possible gene-metabolite links, sometimes resulting in false positives.

Identifying metabolites that differ significantly between case and control groups is critical for discovering disease biomarkers. In this perspective, the ML-based methods outperformed reference-based tools, though all methods faced challenges in detecting differential metabolites with high precision. This limitation in prediction accuracy highlights the complexity of capturing relative abundance differences solely from sequencing data and suggests that further improvements in ML model training data could enhance the robustness of differential metabolite predictions.

### Challenges and limitations in predictive metabolomics

Predictive metabolomics holds promise but faces challenges. Reference-based methods are limited by the coverage and quality of biochemical databases like KEGG and BioCyc, which lack comprehensive data on microbial metabolism, especially for uncultured organisms (Altman et al., 2013). This may lead to missed novel metabolites and interactions in microbiome studies. ML-based approaches depend on the diversity and quality of training data, with higher accuracy seen in datasets that match training conditions. Expanding datasets to cover diverse microbiomes and environments can improve predictions. Additionally, validation of predicted metabolites is challenging due to biases in mass spectrometry and nuclear magnetic resonance (NMR) techniques (Christians et al., 2011; Vuckovic, 2012). Improved databases and validation methods are essential for advancing predictive metabolomics, which could become integral to microbiome research as technologies advance.

## Functional Pathway Analysis Tools for Microbiome Data

The analysis of functional pathways within microbial communities has become a cornerstone in microbiome research, offering insights into metabolic capabilities, ecological roles, and contributions to biogeochemical cycles. A range of computational tools has been developed to facilitate the prediction and profiling of microbial functions from sequencing data. Among these, Phylogenetic Investigation of Communities by Reconstruction of Unobserved States (PICRUSt2) (Douglas et al., 2020), bioBakery (Beghini et al., 2021), and Metabolic And Biogeochemistry analyses in microbes (METABOLIC) (Zhou et al., 2022) stand out as versatile and robust tools, each with unique strengths suited to various aspects of microbiome functional analysis. This section reviews these tools, summarizing their methodologies, key features, and applications. A summary of these tools is also listed in Table 3.

### Phylogenetic inference-based functional prediction

PICRUSt2 was developed as an advancement of PICRUSt1 (Langille et al., 2013), aimed at predicting the functional potential of microbial communities from 16S rRNA gene data. PICRUSt2 enhances the accuracy and flexibility of predictions by integrating phylogenetic placement with an expanded reference database, making it compatible with both operational taxonomic units (OTUs) and amplicon sequence variants (ASVs). This tool can predict gene families and pathway abundances by placing query sequences onto a phylogenetic tree and using a hidden state prediction approach to infer unobserved genomic traits.

The genome database of PICRUS2t includes over 41,000 microbial genomes. This tool has been validated across diverse environments – such as human gut (Parras-Molto & Aguirre de Carcer, 2020), soil (Cupples et al., 2022), and marine microbiomes (Raes et al., 2021) – demonstrating its utility in functional prediction. PICRUSt2 is particularly effective in environments poorly represented in reference databases due to its reliance on phylogenetic methods, which improve prediction accuracy in under-characterized microbial communities.

### Comprehensive meta-omics profiling

bioBakery offers a suite of tools that enable detailed functional and taxonomic profiling of microbial communities using a combination of taxonomic and strain-level profiling tools, such as MetaPhlAn and HUMAnN. MetaPhlAn (together with StrainPhlAn within it) allows for strain-level resolution of community composition, while HUMAnN provides functional profiling by identifying gene families and metabolic pathways directly from metagenomic and metatranscriptomic data. This suite integrates seamlessly to support comprehensive analysis across microbial taxonomic, functional, and strain levels, which is especially useful for distinguishing functional differences within and between microbial populations.

The application of bioBakery to complex datasets, such as those from the human gut, has highlighted its ability to detect specific pathways and gene families relevant to health and disease. For example, bioBakery tools have uncovered functional associations with diseases such as colorectal cancer and IBD (Beghini et al., 2021; Zheng et al., 2024), identifying potential microbial biomarkers and therapeutic targets. Additionally, the open-source and cloud-compatible design of bioBakery makes it widely accessible for large-scale analyses in both research and clinical contexts.

### Functional and biogeochemical network analysis

The METABOLIC toolkit provides a high-throughput framework for profiling metabolic and biochemical functions within microbial communities, with an emphasis on environmental microbiomes. METABOLIC is designed to integrate genome-scale and community-scale analyses, allowing researchers to characterize metabolic networks, detect microbial interactions, and analyze contributions to biogeochemical cycles such as carbon, nitrogen, and sulfur. It uses a combination of KEGG, TIGRfam (Haft et al., 2013), and custom HMMs to annotate microbial genomes for various metabolic functions. Additionally, METABOLIC introduces a motif validation step to improve the specificity of functional predictions, an important feature for accurate biochemical modeling.

METABOLIC has been tested across various ecosystems, including marine and terrestrial environments, freshwater lakes, and human gut microbiomes, showing robust performance in detecting biogeochemically relevant metabolic functions (Chen et al., 2024; Dopson et al., 2024; Li et al., 2024; Northen et al., 2024; Ostos et al., 2024). The toolkit’s compatibility with transcriptomic data further allows for activity-based assessments of microbial functions, making it suitable for both metagenomic and metatranscriptomic studies. By providing a quantitative and visual approach to community-scale interactions, METABOLIC aids researchers in linking microbial functional traits with environmental processes.

### Characteristics and future directions of functional pathway analysis tools

Tools such as PICRUSt2, bioBakery, and METABOLIC represent state-of-the-art tools for functional pathway analysis in microbiome research, each offering unique capabilities suited to different research questions. The phylogenetic placement of PICRUSt2, the comprehensive profiling suite of bioBakery, and the biogeochemical and network analyses of METABOLIC collectively address key challenges in microbial functional inference. Future developments in these tools may include expanded databases, improved algorithms for strain-level resolution, and more advanced integration of multi-omic data, which will further enhance the accuracy and scope of microbiome functional analyses.

## Limitations and Challenges of Current Approaches

Despite the significant advancements in microbiome functional analysis, several limitations and challenges remain, particularly concerning the accuracy and applicability of current computational approaches. Addressing these limitations is crucial for improving the reliability and utility of GEMs, predictive metabolomics, and functional pathway analysis tools.

### Model accuracy and assumptions

One of the central challenges in GEMs is the reliance on the steady-state assumption inherent in FBA. This assumption simplifies the system by focusing on the balance of metabolic fluxes without accounting for dynamic changes in metabolite concentrations over time. While this enables efficient computation, it can lead to inaccuracies when modeling dynamic biological processes, such as growth under fluctuating environmental conditions (Gonzalez & Aranda, 2023; Nhu et al., 2020). dFBA attempts to address this by incorporating temporal dynamics, but it requires more computational resources and detailed knowledge of initial metabolite concentrations.

ML-based predictive metabolomics models offer a data-driven approach to metabolite prediction but are limited by the availability and quality of paired microbiome-metabolome datasets. These models may exhibit overfitting or reduced performance when applied to datasets with different environmental or biological conditions, highlighting the need for diverse and comprehensive training datasets. Moreover, these approaches often lack mechanistic interpretability, which poses challenges for understanding the underlying biological processes.

### Dependence on reference databases

The accuracy of reference-based methods in both GEMs and metabolite prediction depends heavily on the completeness and quality of biochemical databases such as KEGG and BioCyc. However, these databases are known to have gaps, especially for less characterized microbial species and ecosystems (Thiele & Palsson, 2010). This limitation can result in incomplete or inaccurate predictions, particularly in studies involving novel or uncultured microorganisms.

For example, while tools like MIMOSA2 and Mangosteen utilize curated reaction networks, their reliance on existing gene-metabolite associations limits their predictive power in environments with significant microbial diversity (Shtossel et al., 2024). Expanding and curating these databases is essential for improving the scope and reliability of functional predictions.

### Validation and generalizability

Empirical validation remains a bottleneck in the development of computational models. While predictive models can generate hypotheses about microbial functions and interactions, these predictions must be validated through experimental approaches such as targeted metabolomics or isotope-labeling experiments. However, biases in mass spectrometry and NMR techniques used for metabolite validation introduce additional layers of complexity and uncertainty (Marshall & Powers, 2017).

Moreover, the generalizability of current models to different ecosystems and host-microbiome interactions is limited. For instance, models optimized for human gut microbiota may not perform as well in soil or marine microbiomes due to differences in metabolic capabilities and environmental conditions. Developing ecosystem-specific models and incorporating environmental metadata into computational analyses are potential strategies to overcome this challenge.

### Integration of multi-omic data

The integration of multi-omic data (e.g., metagenomics, metatranscriptomics, proteomics, and metabolomics) holds great promise for enhancing the accuracy and depth of microbiome functional analysis. However, current methods often face significant challenges when combining these heterogeneous data types. Differences in data resolution, scale, and experimental protocols can lead to inconsistencies that hinder effective integration and interpretation. Moreover, many existing approaches generate extensive lists of disease-associated features without adequately leveraging the multi-layered structure of the data or offering interpretable, systems-level insights into microbiome-disease interactions. To address these challenges, frameworks like MintTea (Muller et al., 2024) introduce intermediate integration that capture cross-omic dependencies while pursuing robustness and interpretability. However, despite these advancements, the field still requires standardized pipelines and computational frameworks to further enhance the reproducibility and scalability of multi-omic integration. Ensuring robust cross-study validation and addressing confounding factors, such as diet or medication, remain key priorities for advancing multi-omic research.

## Conclusion

The rapid development of computational and high-throughput sequencing technologies has transformed our ability to analyze the microbiome beyond traditional taxonomic profiling, enabling a deeper understanding of the functional potential of microbial communities. GEMs, metabolite prediction algorithms, and pathway analysis tools have become essential in uncovering the metabolic activities, interspecies interactions, and host-microbiome crosstalk that underlie diverse ecological and clinical phenomena. By integrating NGS data with GEMs, researchers can now simulate microbial and community-level metabolism, providing novel insights into how dietary, environmental, and therapeutic interventions impact microbial dynamics and host health.

Predictive metabolomics and functional pathway analysis have further broadened the scope of microbiome research, allowing for the indirect inference of metabolite profiles and pathway activities, even in the absence of direct metabolomics data. These methods highlight the promise of microbiome-based biomarkers for disease diagnosis, therapeutic monitoring, and personalized intervention strategies. ML techniques, especially, are demonstrating potential in metabolite prediction by capturing complex, data-driven relationships that are challenging to identify through traditional, reference-based approaches.

The clinical applications of microbiome functional analysis are also expanding rapidly, offering new avenues for diagnosing, monitoring, and treating various diseases. GEMs have been instrumental in elucidating microbiome-related metabolic shifts in conditions like type 2 diabetes, IBD, and CD (Beura et al., 2024; Fernandes et al., 2023; Zheng et al., 2024). Functional insights have enabled the identification of disease-specific biomarkers, such as increased acetate and butyrate production in metabolic disorders and proinflammatory bacterial species in IBD (Proffitt et al., 2022; Zheng et al., 2024). Microbiome-based diagnostics, including multiplex droplet digital PCR for IBD, have shown promise as noninvasive tools with high sensitivity and specificity (Zheng et al., 2024). Additionally, fecal microbial transplants targeting dysbiosis have been explored for managing ulcerative colitis, though persistent colonization by resistant strains remains a challenge (Zhang et al., 2024). These are the examples of clinical advancements utilizing microbiome functional analysis in developing personalized therapeutic strategies and improving clinical outcomes.

Despite these advancements and clinical benefits, several challenges remain. Reference-based methods are limited by the completeness and quality of biochemical databases, and predictive models face the hurdle of accurately capturing the vast diversity of microbial functions, particularly in under-characterized ecosystems. The continued improvement of biochemical databases and expansion of training datasets for ML will be crucial in overcoming these limitations. Additionally, validation of predictive models with empirical data remains essential to enhance the robustness and applicability of these computational tools.

In conclusion, the integration of GEMs, predictive metabolomics, and pathway analysis tools with microbiome sequencing data is reshaping our understanding of microbial ecology and its relationship with health and disease. As these technologies evolve, they hold significant potential for advancing precision medicine, enabling the design of targeted dietary and pharmacological interventions tailored to individual microbiomes. The continued synergy between computational innovation and experimental validation will be key to fully realizing the translational impact of microbiome functional analysis in clinical and ecological settings.

## Figures and Tables

**Table 1.** Overview of GEM tools and resources

Name	Purpose/Functionality	Key features
AGORA2 (Heinken et al., 2023)	Personalized and predictive modeling	Models of 7,302 microbial strains
	Model repository	Information on 98 drugs and relevant enzymes
BacArena (Bauer et al., 2017)	Individual-based metabolic modeling of microbial communities	Integrates FBA with individual-based modeling
		Modeling spatial and temporal dynamics
BiGG (King et al., 2016)	Repository for GEMs	77 manually curated GEMs
	Knowledge integration	Supporting various model formats
	Community collaboration	Supporting web API
CarveMe (Machado et al., 2018)	Fast reconstruction of GEMs for microbial species and communities	Top-down approach using a universal model for scalable model generation
		Automated gap-filling for improved growth phenotype predictions
COBRA (Heirendt et al., 2019)	Constraint-based modeling of biochemical networks	Extensive support for FBA and omics data integration
		High-performance solvers for multi-scale and genome-scale models
COMETS (Dukovski et al., 2021)	Dynamic simulation of microbial community interactions	Spatially structured dFBA
		Supports Python and MATLAB interfaces for customized simulations
DyMMM (Zhuang et al., 2011)	Simulating interactions and competition in microbial communities under dynamic conditions	Integrates genome-scale models for multi-species interactions
		Predicts community dynamics under varying environmental conditions
jQMM (Birkel et al., 2017)	Modeling microbial metabolism and analyzing omics data	Combines FBA and 13C metabolic flux analysis
		Uses 13C labeling data for genome-scale model constraints
KBase (Arkin et al., 2018)	Data sharing, integration, and analysis for systems biology	Diverse data integration (genomes, biochemistry)
		Web-based interface with data provenance
MCM (Louca & Doebeli, 2015)	Modeling multi-species microbial communities with genome-based metabolic models	Statistical parameter calibration with experimental data
		dFBA for metabolic interaction simulation
metaGEM (Zorrilla et al., 2021)	Reconstruction of GEMs from metagenome	End-to-end pipeline for community-level metabolic interaction simulations
		Generates personalized metabolic models from metagenome-assembled genomes (MAGs)
MetExplore (Cottret et al., 2018)	Collaborative curation and exploration of metabolic networks	Data mapping for multi-omics integration
		Sub-network extraction and interactive visualization
Microbiome Modeling Toolbox (Heinken & Thiele, 2022)	Efficient modeling and analysis of microbiome communities	Parallelized generation of personalized microbiome models
		Visualization and statistical analysis for model comparison
MMinte (Mendes-Soares et al., 2016)	Predicts metabolic interactions among microbial species in a community	Pairwise interaction analysis under different metabolic conditions
		Modular interface with independent functionalities for flexibility
ModelSEED (Henry et al., 2010)	High-throughput generation and optimization of GEMs	Automated reconstruction pipeline from genome annotation to draft models
		Integrates gap-filling for biomass production and growth simulation
OptCom (Zomorrodi & Maranas, 2012)	Multi-level optimization for modeling metabolic interactions in microbial communities	Balances individual VS. community fitness criteria
		Captures various interaction types (positive, negative) for multiple species
RAVEN (Wang et al., 2018)	Reconstruction and analysis of GEMs	Supports de novo model reconstruction using KEGG and MetaCyc databases
		Integration with COBRA Toolbox for compatibility and bi-directional model conversion
SteadyCom (Chan et al., 2017)	Predicting microbial community composition and maintaining steady-state growth	Ensures constant community growth rate across all species
		Supports flux variability analysis to explore metabolic flexibility
VMH (Noronha et al., 2019)	Integration of models with extrinsic factors such as nutrition and disease	Extensive data coverage (Recon3D human model, 818 microbial models, disease/nutrition information)

**Table 2.** Comparison of metabolite prediction methods

Method type	Name	Data requirements	Advantages	Limitations
ML-based	LOCATE (Shtossel et al., 2024)	Paired microbiome (16S or metagenomics) and metabolomics data	Latent representation and low data requirement for training	Limited cross-dataset generalization
Reference-based	Mangosteen (Yin et al., 2020)	Microbiome sequencing data	Utilizes curated databases	Limited by database coverage
ML-based	MelonnPan (Mallick et al., 2019)	Amplicon or metagenomic sequencing data, paired with metabolomic data for training	Predicts metabolomic profiles from metagenomic data	Requires training data and limited generalization
ML-based	MiMeNet (Reiman et al., 2021)	Paired microbiome (metagenomic taxonomic/functional) and metabolome data	Improves prediction via multivariate learning	Performance depends on dataset size
Reference-based	MIMOSA2 (Noecker et al., 2022)	Paired microbiome (16S or metagenomics) and metabolomics data	Infers mechanistic microbe-metabolite links	Limited to environments represented in reference databases

**Table 3.** Functional pathway analysis tools for microbiome research

Name	Approach	Input data	Unique features
bioBakery (Beghini et al., 2021)	Reference-based, assembly-independent profiling	Metagenomic and metatranscriptomic sequences	Integrates taxonomic, strain-level, functional, and phylogenetic profiling
METABOLIC (Zhou et al., 2022)	High-throughput metabolic and biogeochemical profiling	Genomes from isolates, metagenome-assembled genomes, or single-cell genomes	Community-scale functional networks
MintTea (Muller et al., 2024)	Identification of multi-omic modules	Taxonomic, Functional, Metabolome profiles	Integration of multi-modal data and identifying predictive modules
PICRUSt2 (Douglas et al., 2020)	Phylogenetic placement and hidden state prediction	16S rRNA gene sequences	ASV compatibility, supports custom databases
